# Effects of calcium-magnesium carbonate and calcium-magnesium hydroxide as supplemental sources of magnesium on ruminal microbiome

**DOI:** 10.1093/tas/txac092

**Published:** 2022-07-07

**Authors:** Jose A Arce-Cordero, Ting Liu, Anay Ravelo, Richard R Lobo, Bruna C Agustinho, Hugo F Monteiro, Kwang C Jeong, Antonio P Faciola

**Affiliations:** Department of Animal Sciences, University of Florida, Gainesville, FL 32611, USA; Escuela de Zootecnia, Universidad de Costa Rica, San Jose, 11501-2060, Costa Rica; Emerging Pathogens Institute, University of Florida, Gainesville, FL 32611, USA; Department of Animal Sciences, University of Florida, Gainesville, FL 32611, USA; Department of Animal Sciences, University of Florida, Gainesville, FL 32611, USA; Department of Animal Sciences, University of Florida, Gainesville, FL 32611, USA; Department of Animal Sciences, University of Florida, Gainesville, FL 32611, USA; Emerging Pathogens Institute, University of Florida, Gainesville, FL 32611, USA; Department of Animal Sciences, University of Florida, Gainesville, FL 32611, USA

**Keywords:** alkalizer, butyrate, in vitro, minerals

## Abstract

Our objective was to evaluate the inclusion of calcium-magnesium carbonate [CaMg(CO_3_)_2_] and calcium-magnesium hydroxide [CaMg(OH)_4_] in corn silage-based diets and their impact on ruminal microbiome. Our previous work showed a lower pH and molar proportion of butyrate from diets supplemented with [CaMg(CO_3_)_2_] compared to [CaMg(OH)_4_]; therefore, we hypothesized that ruminal microbiome would be affected by Mg source. Four continuous culture fermenters were arranged in a 4 × 4 Latin square with the following treatments defined by the supplemental source of Mg: 1) **Control** (100% MgO, plus sodium sesquicarbonate as a buffer); 2) **CO**_**3**_ [100% CaMg(CO_3_)_2_]; 3) **OH** [100% CaMg(OH)_4_]; and 4) **CO**_**3**_**/OH** [50% Mg from CaMg(CO_3_)_2_, 50% Mg from CaMg(OH)_4_]. Diet nutrient concentration was held constant across treatments (16% CP, 30% NDF, 1.66 MCal NEl/kg, 0.67% Ca, and 0.25% Mg). We conducted four fermentation periods of 10 d, with the last 3 d for collection of samples of solid and liquid digesta effluents for DNA extraction. Overall, 16 solid and 16 liquid samples were analyzed by amplification of the V4 variable region of bacterial 16S rRNA. Data were analyzed with R and SAS to determine treatment effects on taxa relative abundance of liquid and solid fractions. Correlation of butyrate molar proportion with taxa relative abundance was also analyzed. Treatments did not affect alpha and beta diversities or relative abundance of phylum, class and order in either liquid or solid fractions. At the family level, relative abundance of *Lachnospiraceae* in solid fraction was lower for CO_3_ and CO_3_/OH compared to OH and Control (*P* < 0.01). For genera, abundance of *Butyrivibrio* (*P* = 0.01) and Lachnospiraceae ND3007 (*P* < 0.01) (both from Lachnospiraceae family) was lower and unclassified Ruminococcaceae (*P* = 0.03) was greater in CO_3_ than Control and OH in solid fraction; while abundance of *Pseudobutyrivibrio* (*P* = 0.10) and Lachnospiraceae FD2005 (*P* = 0.09) (both from Lachnospiraceae family) and *Ruminobacter* (*P* = 0.09) tended to decrease in CO_3_ compared to Control in liquid fraction. Butyrate molar proportion was negatively correlated to Ruminococcaceae (*r* = –0.55) in solid fraction and positively correlated to *Pseudobutyrivibrio* (*r* = 0.61) and Lachnospiraceae FD2005 (*r* = 0.61) in liquid. Our results indicate that source of Mg has an impact on bacterial taxa associated with ruminal butyrate synthesis, which is important for epithelial health and fatty acid synthesis.

## INTRODUCTION

Supplemental sources of Mg are alkaline substances that may aid with control of ruminal pH. Previous research has demonstrated the effectiveness of MgO at increasing ruminal pH and preventing reductions in fiber digestibility and milk fat synthesis in dairy cows (Erdman et al., 1982; Bach et al., 2018). Other sources of Mg have been less studied than MgO; however, some recent evaluations indicate that alternative sources of Mg may allow for similar performance of dairy cows when compared to MgO (Leno et al., 2017; Lobo et al., 2021).

Specifically for ruminal fermentation, we recently reported that supplementing corn silage-based diets with either MgO or an alternative source of Mg consisting of a blend of calcium-magnesium carbonate [CaMg(CO_3_)_2_] and calcium-magnesium hydroxide [CaMg(OH)_4_] allowed for similar pH and ruminal fermentation in continuous culture (Arce-Cordero et al., 2021b). Furthermore, in our companion study in continuous culture fermenters evaluating the effect of supplemental sources of Mg on ruminal fermentation (Arce-Cordero et al., 2021a), we found using CaMg(CO_3_)_2_ as the sole source of supplemental Mg allowed for a more acidic ruminal fermentation pattern, as indicated by lower ruminal pH and lower molar proportion of butyrate in comparison to diets formulated with either CaMg(OH)_4_ alone or combined with CaMg(CO_3_)_2_.

To date, we are not aware of any studies evaluating the impact of Mg sources on ruminal microbiota. Therefore, our objective was to evaluate the effects of CaMg(CO_3_)_2_ and CaMg(OH)_4_ as supplemental Mg sources on ruminal microbiota in a dual-flow continuous culture. Based on the results of our companion study (Arce-Cordero et al., 2021a), we hypothesized that ruminal microbiome would be affected by Mg source; more specifically, supplementing CaMg(CO_3_)_2_ as the sole source of supplemental Mg would decrease the relative abundance of bacterial taxa associated with ruminal synthesis of butyrate compared to a diet formulated with either CaMg(OH)_4_ alone or combined with CaMg(CO_3_)_2_.

## MATERIALS AND METHODS

### Experimental Design and Diets

All procedures for care and handling of animals required for this experiment were approved by University of Florida’s Institutional Animal Care and Use Committee. A detailed description of the experimental design and diets can be found in our companion study (Arce-Cordero et al., 2021a). Briefly, four fermenters of a dual-flow continuous culture system were arranged in a 4 × 4 Latin square design with treatments defined by the main supplemental source of Mg added to a common basal diet (Table 1). Treatments were: 1) Control: 100% supplemental Mg from MgO, plus 0.6% sodium sesquicarbonate (to represent the most commonly fed source of Mg which is normally fed along with sodium based buffer); 2) CO_3_: 100% supplemental Mg from CaMg(CO_3_)_2_, without buffer; 3) OH: 100% supplemental Mg from CaMg(OH)_4_, without buffer; and 4) CO_3_/OH: 50% supplemental Mg from CaMg(CO_3_)_2_ and 50% from CaMg(OH)_4_, without buffer. All diets were formulated to provide the same concentration of nutrients regardless of treatment and according to the NRC (2001) recommendations for lactating Holstein cows with 680-kg body weight, and milk production of 45 kg per day with 3.5% fat, 3.0% protein, and 4.8% lactose.

**Table 1.** Ingredient and chemical composition of experimental diets

Item	Treatment^1^			
	Control	CO_3_	OH	CO_3_/OH
Item, % DM				
Corn silage	34.92	35.10	35.14	35.12
Ground corn	33.31	33.48	33.51	33.49
Soybean meal	18.61	18.71	18.73	18.72
Grass hay	9.80	9.85	9.86	9.85
CaCO_3_	1.34	1.18	1.18	1.18
White salt	0.49	0.49	0.49	0.49
Trace mineral premix	0.49	0.49	0.49	0.49
Calcium phosphate	0.34	0.34	0.34	0.34
Sodium sesquicarbonate	0.60	–	–	–
MgO	0.10	–	–	–
CaMg(CO_3_)_2_	–	0.35	–	0.18
CaMg(OH)_4_	–	–	0.26	0.13
Chemical composition, %DM^2^				
CP	16.1	16.2	16.2	16.2
NDF	30.3	30.5	30.5	30.5
Starch	33.1	33.3	33.3	33.3
NEl, Mcal/kg	1.66	1.66	1.67	1.66
Mg	0.25	0.25	0.25	0.25

Experimental treatments based on supplemental source of Mg: “**Control**” = 100% as MgO + sodium sesquicarbonate added as a buffer; “**CO**_**3**_” = 100% as CaMg(CO_3_)_2_; “**OH**” = 100% as CaMg(OH)_4_; “**CO**_**3**_**/OH**” = 50% as CaMg(CO_3_)_2_/ 50% as CaMg(OH)_4_.

Expressed as percent of DM unless otherwise stated.

Ingredients and chemical composition of experimental diets are presented in Table 1. Corn silage was dried for 72 h at 60 °C in a forced-air oven (Heratherm, Thermo Scientific, Waltham, MA) and all ingredients were ground to 2-mm particle size in a Wiley mill (model N°2; Arthur H. Thomas Co., Philadelphia, PA). One sample of each feed was further ground to 1-mm particle size for chemical analyses.

### Dual-flow Continuous Culture System Operation

For this experiment, we used a dual-flow continuous culture based on the system originally developed by Hoover et al. (1976) as described by Brandao et al. (2020) and Arce-Cordero et al. (2020). In this system, ruminal fermentation was simulated through continuous agitation (100 rpm), infusion of N_2_ gas to displace oxygen, constant temperature (39 °C), and infusion of artificial saliva (Weller and Pilgrim, 1974) with 0.40 g/L of urea, at 3.05 mL per min to individually regulate passage rates of liquid (11% h^−1^) and solid (5.5% h^−1^) effluents of digesta.

This experiment consisted of four fermentation periods of 10 d each (40 d of fermentation total). Fermenters were inoculated on day 1 of each fermentation period with fresh ruminal contents collected from two cannulated Holstein cows in midlactation (108 ± 9 DIM on average) that were maintained throughout the experiment and fed twice a day a total mixed ration with 38% corn silage, 19% ground corn, 13% soybean meal, 11% cottonseed, 9% citrus pulp, 8.5% mineral premix, and 1.5% palmitic acid supplement (on a DM basis) from 3 wk before start and until completion of the experiment. Approximately 1 h after morning feeding, ruminal contents were manually collected from each cow and strained through 2 layers of cheesecloth, transferred into prewarmed thermos jars, and immediately transported to the lab. Each fermenter was prewarmed and under continuous flush of N_2_ gas at the moment of inoculation when it was filled with a 50:50 mix (v/v) of ruminal contents from both cows.

Each fermenter was provided its corresponding experimental diet (106 g DM d^−1^) distributed into two portions of 53 g DM at 0800 and 1800 hours. Throughout days 8, 9, and 10 of fermentation, containers of solid and liquid digesta effluent were kept in an ice-cold water bath and digesta temperature maintained –2 °C to prevent any further microbial fermentation of nutrients happening outside of the fermenters. On day 10 upon completion of each period, all fermenters were put apart and cleaned, disposable components of the system were replaced, and fermenters were re-assembled, and re-randomized into experimental treatments for the following period.

### Collection of Data and Samples

First 7 d of fermentation of each period were used for adaptation to experimental diets and stabilization of bacterial communities (Salfer et al., 2018). Collection of data and samples were performed on days 8, 9, and 10 of each period. Results on main fermentation variables such as pH, VFA, and ammonia nitrogen have been reported in our companion study (Arce-Cordero et al., 2021a).

Samples for bacterial sequencing analysis were collected from both liquid and solid effluents of each fermenter every day at 3, 6, and 9 h after morning feed provision. For the liquid fraction, 15 mL of liquid effluent were collected at each timepoint, totaling 45 mL per fermenter per day. For the solid fraction, 200 g of solid effluent were collected at each timepoint and strained through four layers of cheesecloth, totaling an approximate of 25 g of solid sample collected from each fermenter per day. Upon collection, samples were stored at –80 °C for subsequent DNA extraction.

At the end of each day of fermentation, samples were collected for butyrate analysis as reported in our companion study (Arce-Cordero et al., 2021a). Data of butyrate molar proportion were used for analysis of correlation with relative abundance of bacterial taxa.

### Chemical Composition of Feed Ingredients

Samples of feed ingredients for experimental diets were analyzed for: DM (AOAC, 1990; method 930.15), ash (AOAC, 1990; method 942.05), NDF (Van Soest et al., 1991) adapted for Ankom^200^ Fiber Analyzer (Ankom Technology, Macedon, NY) with heat-stable α-amylase and sodium sulphite, total N (AOAC, 2000; method 990.03) by rapid combustion with a micro elemental N analyzer (Vario Micro Cube, Elementar, Hanau, Germany), total starch by enzymatic hydrolysis (AOAC, 2000; method), and Ca, P, and Mg by inductively coupled plasma mass spectrometry (AOAC, 2000; method 985.01).

### DNA Extraction

Samples were thawed at room temperature and combined across days and timepoints within the same period and fermenter, resulting in 16 samples of liquid effluent fraction and 16 samples of solid effluent fraction that were processed individually for analysis. Genomic DNA of liquid and solid effluent samples were extracted separately according to the methodology of Stevenson and Weimer (2007) and described by Arce-Cordero et al. (2022) for samples of continuous culture fermenters. In the case of solid samples, 22 g were blended with extraction buffer (Tris–HCl, ethylenediaminetetraacetic acid, and NaCl) and centrifuged at 500 × *g* for 15 min at 4 °C Samples of liquid fraction and resulting supernatant from solid samples were processed equally, by centrifuging 22 mL at 10,000 × *g* for 25 min at 4 °C and resuspending the bacterial pellet in DNA extraction buffer.

Bacterial pellets were added 20% sodium lauryl sulfate solution and phenol and processed in a bead beater machine (Biospec Products) using zirconium beads (BioSpec Products, Bartlesville, OK). Then, DNA extraction was performed through sequential centrifugations with phenol, phenol/chloroform, and chloroform; and precipitated with 3 M sodium acetate buffer and isopropanol. Resulting DNA was centrifuged with 70% ethanol and the pellet was resuspended in Tris–EDTA buffer. Following extraction, DNA concentration was measured with a Qubit Fluorometer (Invitrogen, San Diego, CA) and stored at –80 °C.

### DNA Amplification and Sequencing

The V4 variable region of bacterial 16S rRNA gene was amplified using dual-index primers (Caporaso et al., 2011) according to Kozich et al. (2013). The PCR amplification reaction consisted of 1 µL forward index primer (10 mM), 1 µL reverse index primer (10 mM), 1 µL DNA template (10 ng/µL), and 17 µL Pfx AccuPrime master mix (Invitrogen, USA). The protocol for the reaction was the following: denaturation for 5 min at 95 °C, followed by 30 cycles of 95 °C for 30 s, annealing at 55 °C for 30 s, extension at 72 °C for 1 min, and elongation for 5 min at 72 °C. Resulting amplicons were run on a 1% agarose gel to confirm success of the PCR and normalized with a SequalPrep Normalization Plate Kit (Applied Biosystems, Foster City, CA) for construction of the DNA pool library. Overall, 32 samples (16 samples of the liquid effluent fraction and 16 samples of the solid effluent fraction) were sequenced at the Interdisciplinary Center for Biotechnology Research (ICBR) of the University of Florida using a MiSeq reagent kit V2 (2 × 250 cycles run; Illumina, San Diego, CA, USA) in an Illumina MiSeq platform (Illumina, San Diego, CA, USA). The 16S rRNA gene amplicon sequencing data were deposited into the NCBI database (accession number PRJNA811164).

### Bacterial Sequence Data Analysis

Sequencing data were analyzed with a Quantitative Insights into Microbial Ecology (version 2; QIIME 2) pipeline (Bolyen et al., 2019). Paired-end raw reads were imported and quality of the initial bases was evaluated with the Interactive Quality Plot. Divisive Amplicon Denoising Algorithm (DADA2) pipeline implemented in QIIME 2 was used for sequence quality control including steps for filtering low quality reads, denoising reads, merging paired-end reads, and removing chimeric reads. The align-to-tree-mafft-fasttree pipeline from the q2-phylogeny plugin of QIIME 2 was used to generate the phylogenetic tree. Sequencing depth was normalized to 10,800 sequences per sample and the number of amplicon sequence variants (ASVs), richness (Chao1), diversity (Shannon index), and Bray-Curtis distance were calculated by the core-metrics-phylogenetic method. The resulting ASVs were classified into phylum, class, order, family, and genus, using the q2-feature-classifier plugin of QIIME 2 and the SILVA 138 database (https://www.arb-silva.de/documentation/release-1381/). For our analysis, we only considered average relative abundances greater than 0.1% across all samples.

### Statistical Analysis

Data were analyzed with R and SAS 9.4 (SAS Institute Inc., Cary, NC). Results of bacterial community structure (Bray-Curtis distance) were analyzed with R vegan package (Callahan et al., 2016) and visualized by plots of principal coordinate analysis (PCoA). Effects of treatments in community structures were determined with PERMANOVA test implemented in QIIME 2. Relative abundance and alpha diversity data were analyzed with the MIXED procedure of SAS 9.4 (SAS Institute Inc.). The statistical model included treatment as a fixed effect, and random effects of period and fermenter. Correlations between butyrate molar proportion and relative abundance of bacterial taxa affected by treatment were analyzed using the Pearson CORR procedure. Significance was declared at *P* ≤ 0.05, while 0.05 < *P* ≤ 0.10 was considered a trend.

## RESULTS AND DISCUSSION

A total of 32 samples were sequenced consisting of 16 samples of liquid fraction and 16 samples of solid fraction. Overall, 726,628 reads were generated from 16S rRNA sequencing, from which 568,934 high-quality sequences were retained for analysis after filtering, denoising, merging, and removing chimeras with DADA2 pipeline. In total 19 and 20 phyla, 33 and 33 classes, 58 and 63 orders, 88 and 98 families, and 202 and 214 genera were identified across samples of solid and liquid fractions, respectively.

**Figure 1.**
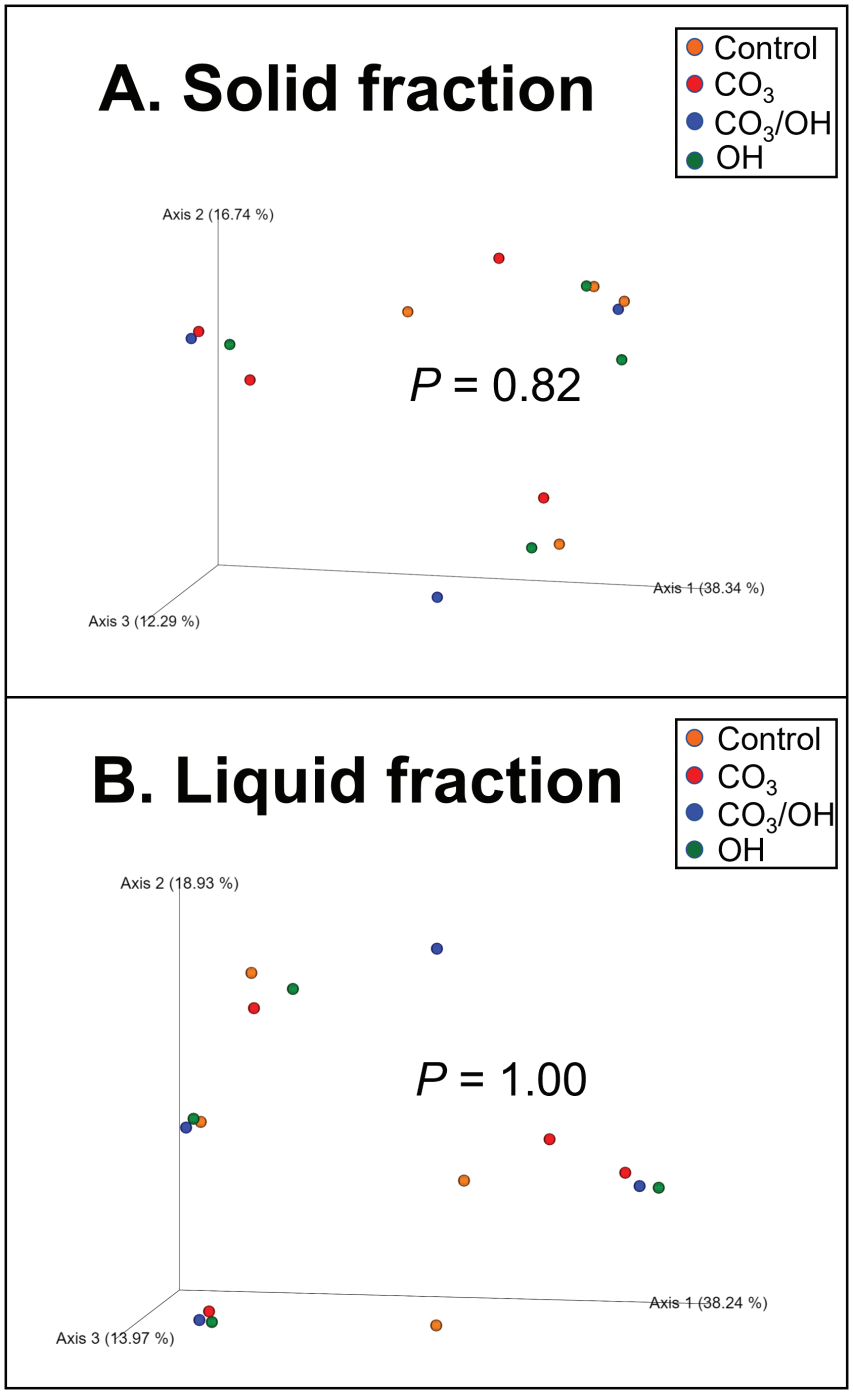
Principal coordinates analysis (PCoA) plots of Bray-Curtis similarity comparing the treatment effects on community structure of ruminal bacteria. Treatments were based on supplemental source of Mg and are denoted as follows: 1) **Control** = 100% as MgO + sodium sesquicarbonate added as a buffer, 2) **CO**_**3**_ = 100% as CaMg(CO_3_)_2_, 3) **OH** = 100% as CaMg(OH)_4_, 4) **CO**_**3**_**/OH** = 50% as CaMg(CO_3_)_2_/ 50% as CaMg(OH)_4_.

**Figure 2.**
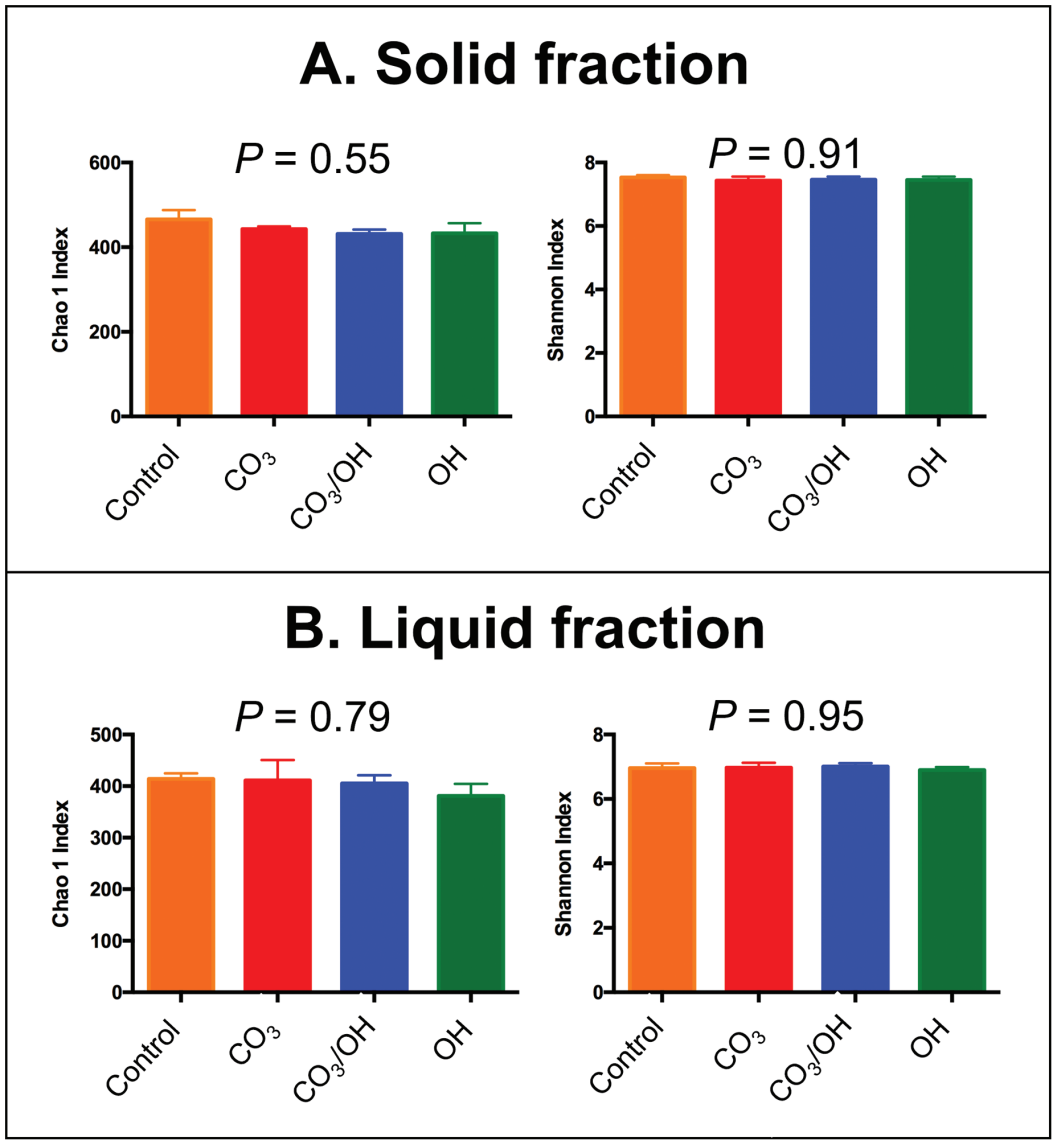
Effects of experimental treatments on alpha diversity of ruminal bacteria. Treatments were based on supplemental source of Mg and are denoted as follows: 1) **Control** = 100% as MgO + sodium sesquicarbonate added as a buffer, 2) **CO**_**3**_ = 100% as CaMg(CO_3_)_2_, 3) **OH** = 100% as CaMg(OH)_4_, 4) **CO**_**3**_**/OH** = 50% as CaMg(CO_3_)_2_/ 50% as CaMg(OH)_4_.

The analyses of treatment effects on bacterial community structure are presented in Figure 1. As observed in panels A (solid fraction) and B (liquid fraction), there were no effects of treatments on bacterial community structure according to Bray–Curtis similarity index. Similarly, for alpha diversity analyzes (Figure 2) we found that treatments did not affect Chao 1 and Shannon indices, indicating that neither richness or evenness of ruminal microbiome, respectively, were affected by treatments in solid (panel A) or liquid (panel B) fractions. In order to better understand the possible effects of CaMg(OH)_4_ and CaMg(CO_3_)_2_ as supplemental Mg sources on ruminal microbiota, we analyzed their effects on relative abundance at multiple taxonomic levels.

At the phylum level (Table 2) in the solid fraction Firmicutes, Bacteroidota, Proteobacteria, Spirochaetota, and Fibrobacterota were the most abundant phyla which accounted on average for 50.3, 22.3, 10.0, 8.7, 3.3% of the sequences, respectively. There were no effects of treatment on phyla relative abundance in the solid fraction. Similarly, relative abundance of phyla in the liquid fraction was not affected by treatment, and averaged 36.9, 34.9, 19.5, 2.3, and 2.1% for Firmicutes, Bacteroidota, Proteobacteria, Actinobacteriota, and Spirochaetota, respectively.

**Table 2.** Effects of calcium magnesium carbonate and calcium magnesium hydroxide on relative abundance of main phyla of bacteria in solid and liquid fractions

Phylum	Treatment means^1^				SEM	*P*-value^2^
	Control	CO_3_	OH	CO_3_/OH		
Solid fraction						
Firmicutes	51.1	49.6	50.9	49.7	1.83	0.87
Bacteroidota	22.6	21.3	21.8	23.7	1.13	0.44
Proteobacteria	9.86	11.2	9.88	9.08	1.66	0.86
Spirochaetota	8.61	7.79	9.28	9.07	1.1	0.66
Fibrobacterota	3.65	2.87	3.30	3.23	0.43	0.53
Liquid fraction						
Firmicutes	36.6	36.8	36.9	37.4	1.74	0.94
Bacteroidota	35.1	33.8	34.3	36.5	2.27	0.75
Proteobacteria	21.0	18.6	20.7	17.6	1.6	0.41
Actinobacteriota	1.10	4.68	1.71	1.56	1.77	0.48
Spirochaetota	2.06	1.90	2.12	2.22	0.34	0.93

Experimental treatments based on supplemental source of Mg: “**Control**” = 100% as MgO + sodium sesquicarbonate added as a buffer; “**CO**_**3**_” = 100% as CaMg(CO_3_)_2_; “**OH**” = 100% as CaMg(OH)_4_; “**CO**_**3**_**/OH**” = 50% as CaMg(CO_3_)_2_/ 50% as CaMg(OH)_4_. Means with different superscript within the same row are statistically different (*P* ≤ 0.05).

Effect of experimental treatment.

Our results indicate that diets supplemented with either CaMg(CO_3_)_2_ or CaMg(OH)_4_ as supplemental sources of Mg do not allow for major changes in ruminal bacterial populations, as indicated by similar bacterial community structure, richness, evenness, and relative abundance of phyla across experimental treatments. The fact that all the diets were formulated to the same concentration of Mg and differed only in the source of supplemental Mg (with the exception of the positive Control treatment that included sodium sesquicarbonate as a buffer that was not included in the three other diets) may have influenced the lack of differences in alpha and beta diversity and relative abundance of phyla across treatments.

Relative abundance of the main families is presented in Table 3. In the solid fraction, we found that Lachnospiraceae was the most abundant group with an average 26.9% relative abundance across experimental treatments, followed by Prevotellaceae, Succinivibrioceae, Spirochaetaceae, and Selenomonadaceae, whose average relative abundances were 18.1%, 9.8%, 8.7%, and 5.3%. More importantly, Lachnospiraceae was the only family whose relative abundance was affected by treatment in the solid fraction, being lower for CO_3_ and CO_3_/OH treatments in comparison to Control and OH. Previous research indicates that Mg requirement by ruminal bacteria should be satisfied by Mg concentrations commonly found in ruminal fluid (Morales and Dehority, 2014). Therefore, it is possible that any effects of Mg supplementation on ruminal microbiota may be a consequence of the impact of Mg sources on ruminal pH rather than a direct effect of Mg source on the microorganisms. In this regard, it is important to point out that Mg sources evaluated in this experiment are salts that differ in the anion accompanying the Ca and Mg cations, which may also play a role in ruminal fermentation and microbial stimulation. The OH− anion is a well-known strong base that may have an impact on pH by counteracting H^+^ cations; on the other hand, $C{O_{3}}^{2-}$ anions can contribute with the bicarbonate buffering system, which, given its chemical nature and greater p*K*a, may be of greater relevance at ranges of pH above the ruminal pH, such as the one in the intestinal environment, where studies have shown that CaCO_3_ supplementation increases intestinal and fecal pH (Tissera et al., 1988).

**Table 3.** Effects of calcium magnesium carbonate and calcium magnesium hydroxide on relative abundance of main families of bacteria in solid and liquid fractions

Family	Treatment means^1^				SEM	*P*-value^2^
	Control	CO_3_	OH	CO_3_/OH		
Solid fraction						
Lachnospiraceae	28.6^a^	25.0^b^	28.4^a^	25.4^b^	1.04	<0.01
Prevotellaceae	18.6	17.1	17.5	19.2	1.00	0.40
Succinivibrionaceae	9.59	10.95	9.66	8.94	1.66	0.87
Spirochaetaceae	8.46	7.84	9.30	9.04	1.10	0.68
Selenomonadaceae	4.26	5.51	4.88	6.50	0.90	0.33
Ruminococcaceae	4.16	5.72	4.31	4.84	0.72	0.11
Acidaminococcaceae	3.58	3.44	3.16	3.13	0.29	0.67
Fibrobacteraceae	3.69	2.89	3.29	3.26	0.44	0.53
Christensenellaceae	2.68	2.34	2.28	2.05	0.36	0.67
Oscillospiraceae	1.78	1.83	1.81	1.61	0.28	0.96
Liquid fraction						
Prevotellaceae	23.0	22.1	21.3	22.1	2.23	0.77
Succinivibrionaceae	20.8	18.9	20.7	17.4	1.66	0.43
Lachnospiraceae	15.3	14.7	15.6	15.5	0.79	0.57
Bacteroidales F082	5.46	5.46	6.87	6.77	2.55	0.30
Selenomonadaceae	3.56	5.31	4.54	4.33	1.08	0.20
Rikenellaceae	4.51	3.50	4.16	4.35	0.61	0.33
Oscillospiraceae	3.97	3.30	3.54	3.31	0.69	0.75
Acidaminococcaceae	3.19	3.19	3.60	2.98	0.36	0.53
Ruminococcaceae	2.80	3.39	2.18	3.90	1.19	0.48
Christensenellaceae	2.15	2.05	2.10	2.21	0.27	0.84

Experimental treatments based on supplemental source of Mg: “**Control**” = 100% as MgO + sodium sesquicarbonate added as a buffer; “**CO**_**3**_” = 100% as CaMg(CO_3_)_2_; “**OH**” = 100% as CaMg(OH)_4_; “**CO**_**3**_**/OH**” = 50% as CaMg(CO_3_)_2_/ 50% as CaMg(OH)_4_. Means with different superscript within the same row are statistically different (*P* ≤ 0.05).

Effect of experimental treatment.

Studies in dairy cows have reported a positive correlation of Lachnospiraceae relative abundance in the rumen with DMI in early lactation (Bach et al., 2019) and feed efficiency in midlactation (Delgado et al., 2019). Previous research on members of family Lachnospiraceae indicates that two of its main genera, *Butyrivibrio* and *Lachnospira*, are present in the rumen and involved with synthesis of volatile compounds such as butyrate and acetate, respectively (Cotta and Forster, 2006). Our companion study showed that CO_3_ had a lower butyrate molar proportion (15.0%) in comparison to Control (16.4%) and CO_3_/OH (16.3%) (Arce-Cordero et al., 2021a), which may have been a consequence of the presence of butyrogenic bacteria within the Lachnospiraceae family, whose relative abundance was lower for CO_3_.

Relative abundance of families in the liquid fraction was not affected by treatment (Table 3). In this case, Prevotellaceae was the most abundant group, with an average 22.1% relative abundance across treatments, followed by Succinivibrioceae, Lachnospiraceae, Bacteroidales F082, and Selenomonadaceae, whose average relative abundances were 19.4%, 15.3%, 6.1%, and 4.4%, respectively. Prevotellaceae has been previously reported as the most abundant family of ruminal bacteria in ruminal fluid samples (Sollinger et al., 2018, Monteiro et al., 2022).

At the genus level, relative abundances in the solid fraction were affected or at least tended to be affected by our experimental treatments (Table 4). Overall, 13 genera were somehow influenced by treatments, eight of them belong to the Lachnospiraceae family, three are in the Prevotellaceae family, one in the *Bacteroidales*, and one in Ruminococcaceae. These results are consistent with those presented in Table 3 showing that our treatments had an influence in relative abundance of Lachnospiraceae family in the solid fraction. Relative abundance of *Butyrivibrio* and Lachnospiraceae ND3007 was lower for CO_3_ in comparison to Control and OH; moreover, abundance of unclassified Lachnospiraceae was lower in CO_3_/OH compared to Control and OH but similar to CO_3_. Additionally, relative abundance of unclassified Prevotellaceae was greater in the CO_3_ and CO_3_/OH treatments than in Control and tended to be greater in CO_3_ than in Control for Prevotellaceae UCG-003. Abundance of unclassified Ruminococcaceae was also greater in CO_3_ compared to the three other treatments. Conversely, Prevotellaceae Ga6A1 tended to be less abundant in CO_3_ compared to Control.

**Table 4.** Effects of calcium magnesium carbonate and calcium magnesium hydroxide on genera relative abundance of bacteria in solid fraction

Genus	Family	Treatment means^1^				SEM	*P*-value^2^
		Control	CO_3_	OH	CO_3_/OH		
*Butyrivibrio*	Lachnospiraceae	3.77^ab^	2.62^cd^	4.46^ab^	3.61^bc^	0.49	0.01
Unclassified	Lachnospiraceae	0.49^ab^	0.32^bc^	0.51^ab^	0.26^cd^	0.05	0.03
*[Ruminococcus] gauvreauii group*	Lachnospiraceae	0.33^bc^	0.41^ab^	0.18^cd^	0.37^bc^	0.09	0.03
*FCS020*	Lachnospiraceae	0.47	0.36	0.22	0.20	0.08	0.09
*Marvinbryantia*	Lachnospiraceae	0.34	0.26	0.17	0.13	0.07	0.09
*ND3007*	Lachnospiraceae	0.23^a^	0.18^b^	0.27^a^	0.13^b^	0.04	<0.01
*[Eubacterium] hallii*	Lachnospiraceae	0.23^ab^	0.18^bc^	0.15^cd^	0.20^bc^	0.04	0.05
*XBB1006*	Lachnospiraceae	0.14^b^	0.08^b^	0.27^a^	0.15^b^	0.03	<0.01
*Ga6A1group*	Prevotellaceae	2.28	1.23	1.98	1.60	0.26	0.08
*UCG-003*	Prevotellaceae	0.47	0.72	0.75	0.57	0.09	0.08
Unclassified	Prevotellaceae	0.11b^c^	0.20^ab^	0.08^cd^	0.19^ab^	0.04	0.02
Unclassified	Ruminococcaceae	0.76^b^	2.82^a^	1.29^b^	1.83^b^	0.52	0.04
*BS11gut group*	Bacteroidales	0.25^cd^	0.37^bc^	0.29^cd^	0.54^ab^	0.1	0.02

Experimental treatments based on supplemental source of Mg: “**Control**” = 100% as MgO + sodium sesquicarbonate added as a buffer; “**CO**_**3**_” = 100% as CaMg(CO_3_)_2_; “**OH**” = 100% as CaMg(OH)_4_; “**CO**_**3**_**/OH**” = 50% as CaMg(CO_3_)_2_/50% as CaMg(OH)_4_. Means with different superscript within the same row are statistically different (*P* ≤ 0.05).

Effect of experimental treatment.

Our findings are consistent with the lower molar proportion of butyrate observed for CO_3_ (15.0%) in comparison to Control (16.4%) and OH (15.8%) in our companion study (Arce-Cordero et al., 2021a). Genus *Butyrivibrio* has been described as a group of butyrate-synthesizing bacteria capable of fermenting a wide variety of carbohydrates but primarily relying on xylan as their main substrate (Cotta and Forster, 2006). On the other hand, studies with some Lachnospiraceae genera indicate their ability to synthesize butyrate (Meehan and Beiko, 2014) which results primarily from degradation of pectins (Cotta and Forster, 2006).

**Figure 3.**
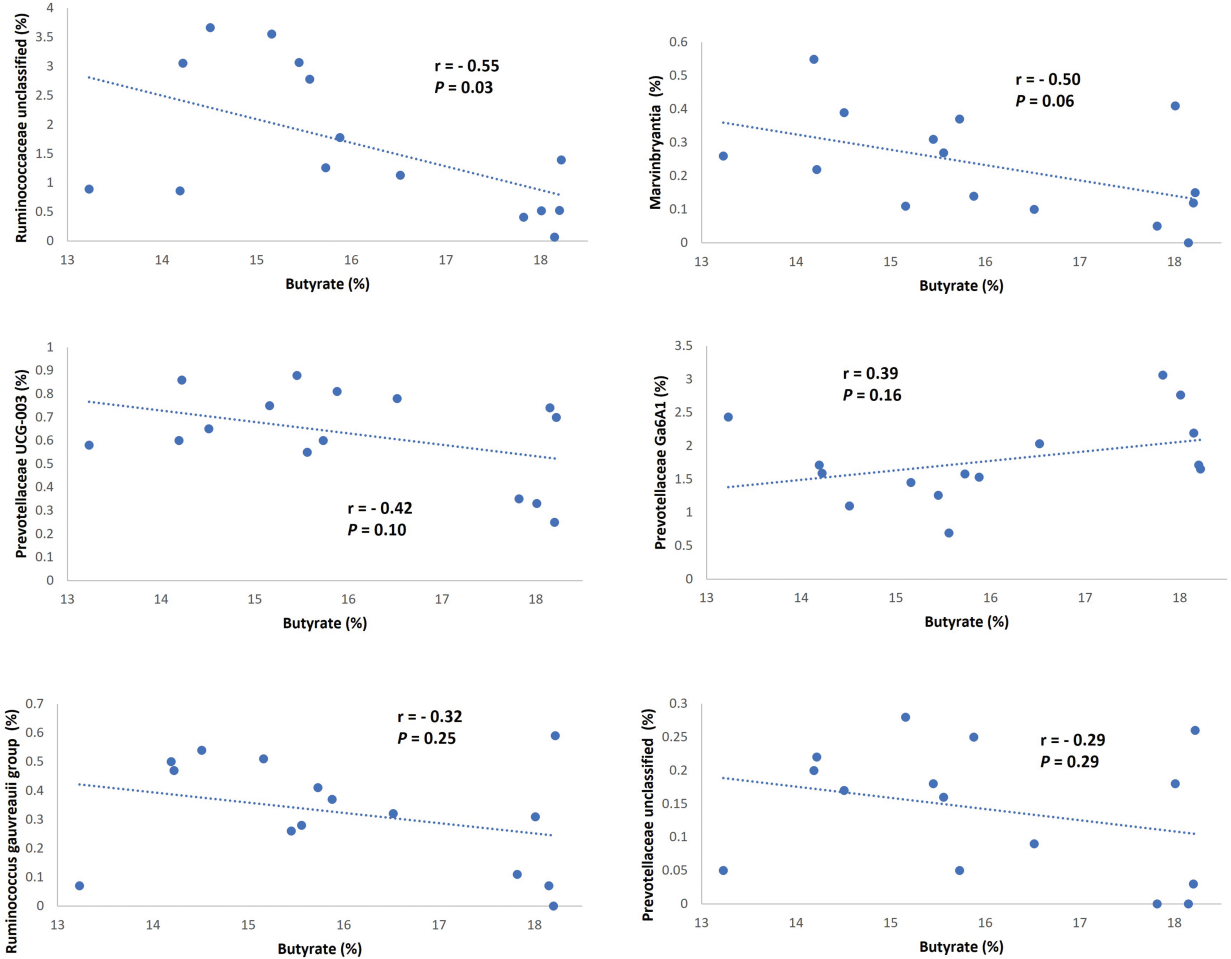
Correlation between molar proportion of butyrate in ruminal fluid and relative abundance of genera in solid fraction. The six correlations with larger Pearson coefficient (|*r*|)are presented.

Average relative abundance of *Butyrivibrio* in solid fraction of the present study averaged 3.6% (Table 4), indicating that a substantial fraction of the bacterial community was influenced by CO_3_. Nevertheless, we did not observe a correlation between relative abundance of *Butyrivibrio* and butyrate molar proportion in the solid fraction. Our correlation analysis between genera abundance and butyrate molar proportion in solid fraction is depicted in Figure 3, showing the six genera with larger Pearson correlation coefficients. Butyrate molar proportion was negatively correlated with relative abundance of unclassified Ruminococcaceae (*r* = –0.55) and tended to correlate negatively with *Marvinbryantia* (*r* = –0.50) and Prevotellaceae UCG-003 (*r* = –0.42).

Greater relative abundance of some genera within the Ruminococcaceae family has been observed in ruminants consuming diets with a greater inclusion of forage, suggesting their involvement in fiber degradation and hence their preference for less acidic ruminal pH (Henderson et al., 2015; Zeng et al., 2019), which does not agree with our results indicating that CO_3_ promotes greater abundance of unclassified Ruminococcaceae, and according to our companion study (Arce-Cordero et al., 2021a), a more acidic pH and lower butyrate molar proportion in comparison to the three other treatments. However, variation in preference for substrates and ruminal conditions seems to be present among members of the Ruminococcaceae family. Bach et al. (2019) reported that the relative abundance of six genera within the Ruminococcaceae family decreased during the period going from –2 to 3 wk from calving in dairy cows; however, the opposite was observed for three other genera of the same family, which confirmed the high functional diversity reported within the Ruminococcaceae family.

Previous research has shown that butyrate synthesis is not exclusive to *Butyrivibrio* and although some members of the Lachnospiraceae family (including genus *Butyrivibrio*) are primarily butyrate producers, other members of this family do not synthesize butyrate as their main fermentation product (Meehan and Beiko, 2014), which may be the reason why we did not observe a correlation between butyrate molar proportion and relative abundance of family Lachnospiraceae or genus *Butyrivibrio* in the solid fraction of our study. Moreover, two main mechanisms have been identified for bacterial synthesis of butyrate within the Lachnospiraceae family, butyryl CoA/acetyl CoA transferase activity and butyrate kinase pathway (Diez-Gonzalez et al., 1999; Cotta and Forster, 2006) the former utilizes acetate as part of the process, while the later produces acetate as a byproduct. The impact of both pathways on the interaction of *Lachnospiraceae* family members with other groups of bacteria involved in ruminal acetate synthesis has not been well established (Cotta and Forster, 2006), which may further challenge the establishment of direct associations between VFA molar proportions and relative abundance of bacteria that belong to this family.

Relative abundance of genera in the liquid fraction is presented in Table 5. As observed, relative abundance of Prevotellaceae UCG-003 was greater in CO_3_ than Control and CO_3_/OH. Moreover, for members of the Lachnospiraceae family, relative abundance of *NK3A20* tended to be greater for CO_3_ compared to Control; however, the opposite trend was observed for *Pseudobutyrivibrio* and *FD2005*. Abundance of genus *Schwartzia* from Selenomonadaceae family tended to be greater for CO_3_ compared to Control; conversely, abundance of *Ruminobacter* belonging to Succinivibrionaceae family tended to decrease with CO_3_ and CO_3_/OH in comparison to Control.

**Table 5.** Effects of calcium magnesium carbonate and calcium magnesium hydroxide on genera relative abundance of bacteria in liquid fraction

Genus	Family	Treatment means^1^				SEM	*P*-value^2^
		Control	CO_3_	OH	CO_3_/OH		
*NK3A20*	Lachnospiraceae	3.71	5.01	4.54	4.52	0.41	0.08
*Pseudobutyrivibrio*	Lachnospiraceae	1.86	1.00	1.61	1.50	0.46	0.10
*FD2005*	Lachnospiraceae	0.64	0.28	0.32	0.46	0.19	0.09
*UCG-003*	Prevotellaceae	1.21^bc^	1.69^ab^	1.55^bc^	1.09^cd^	0.17	0.03
*Schwartzia*	Selenomonadaceae	1.36	1.82	1.60	1.29	0.28	0.07
*Ruminobacter*	Succinivibrionaceae	1.63	1.03	1.99	0.64	0.75	0.09

Experimental treatments based on supplemental source of Mg: “**Control**” = 100% as MgO + sodium sesquicarbonate added as a buffer; “**CO**_**3**_” = 100% as CaMg(CO_3_)_2_; “**OH**” = 100% as CaMg(OH)_4_; “**CO**_**3**_**/OH**” = 50% as CaMg(CO_3_)_2_/ 50% as CaMg(OH)_4_. Means with different superscript within the same row are statistically different (*P* ≤ 0.05).

Effect of experimental treatment.

Members of the Prevotellaceae family (such as genus *UCG-003*) play a role in the synthesis of starch-degrading enzymes in the rumen (Sollinger et al., 2018) and therefore their abundance is greater in diets with a greater concentration of nonstructural carbohydrates and lower ruminal pH. Similarly, members of Selenomonadaceae family such as genus *Schwartzia*, have been associated with ruminal degradation of nonstructural carbohydrates and more acidic ruminal pH (Deusch et al., 2017). This is consistent with the results obtained in our companion study where we observed a greater area under the pH curve (pH × h) for CO_3_ (22.3) compared to Control (7.4), OH (11.9), and CO_3_/OH (6.0). On the other hand, greater relative abundance of bacteria of the Succinivibrionaceae family and their metaproteome have been reported for lactating cows when concentration of nonstructural carbohydrates in the diet is increased (Deusch et al., 2017). Moreover, experiments evaluating substrate utilization have shown that bacteria from genus *Ruminobacter* within the Succinivibrionaceae family are starch degrading microorganisms, with virtually no affinity for glucose or nonstarch polysaccharides (Anderson, 1995). Based on that, we would have expected to observe a greater abundance of *Ruminobacter* in CO_3_ than the three other treatments; however, we found the opposite. Greater abundance of *Ruminobacter* has been reported as a result of butyrate infusion in the rumen of lactating cows (Li et al., 2012), suggesting a possible relationship between *Ruminobacter* and butyrate; however, the mechanisms or bacterial interactions behind it are not clear.

**Figure 4.**
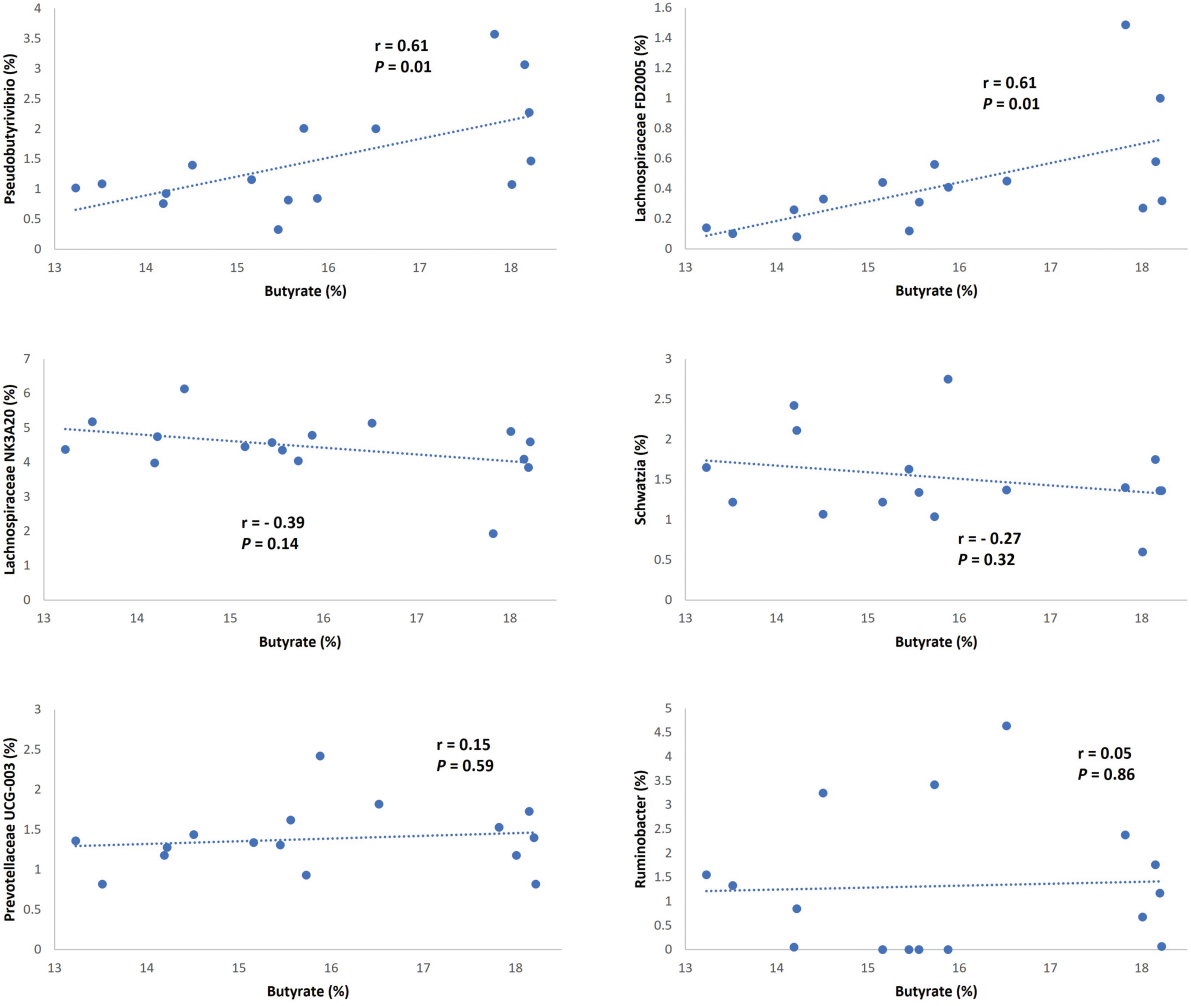
Correlation between molar proportion of butyrate in ruminal fluid and relative abundance of genera in liquid fraction. The six correlations with larger Pearson coefficient (|*r*|) are presented.

The correlation between molar proportion of butyrate and relative abundance of genera in liquid fraction is presented in Figure 4. Our analysis indicated that relative abundances of *Pseudobutyrivibrio* and Lachnospiraceae FD2005 were positively correlated with butyrate molar proportion (*r* = 0.61 for both genera), which is consistent with the reduction in relative abundance of these two genera observed with CO_3_ in comparison to Control. Members of the Lachnospiraceae family are known to play an important role in ruminal synthesis of butyrate (Cotta and Forster, 2006). Moreover, bacteria of genus *Pseudobutyrivibrio* have been shown to encode a large spectrum of carbohydrate active enzymes and binding proteins that are involved in processes of depolymerization and utilization of insoluble plant polysaccharides (Palevich et al., 2020) having butyrate as one of its main products of fermentation (Gylswyk et al., 1996).

Overall, our results indicate that supplemental source of Mg has an impact on ruminal microbiome, particularly on bacterial taxa associated with ruminal synthesis of butyrate, which is important for epithelial health and fatty acid synthesis. Moreover, this research contributes to the current body of knowledge towards a better understanding of the animal response to Mg supplementation.
